# Solitary metastases of colon adenocarcinoma to the ankle: A case report and literature review

**DOI:** 10.1016/j.ijscr.2025.111515

**Published:** 2025-06-14

**Authors:** Mohammadsadra Shamohammadi, Armaghan Abbasi Garavand, Behnoush Mohammadtaheri, Mahdi Alemrajabi, Seyed Hamzeh Mousavie, Ali Zare Mehrjardi

**Affiliations:** a Gastrointestinal and Liver Diseases Research Center, Iran University of Medical Sciences, Tehran, Iran; b School of Medicine, Iran University of Medical Sciences, Tehran, Iran; c Department of Pathology, Firoozgar General Hospital, Iran University of Medical Sciences, Tehran, Iran

**Keywords:** Colorectal cancer, Solitary bone metastases, Signet ring cell carcinoma, Talus, Case report

## Abstract

**Introduction:**

Colorectal cancer (CRC) is a major cause of cancer-related mortality, with metastases typically involving the liver and lungs. Solitary bone metastasis to the ankle is exceedingly rare and presents significant diagnostic challenges.

**Case presentation:**

A 46-year-old male presented with constipation, abdominal pain, and persistent right foot discomfort following a previous talus fracture that failed to heal with conservative management. Imaging studies revealed a sigmoid colon mass and a Solitary lesion in the right talus. Biopsy of the colon and ankle specimens confirmed poorly differentiated signet ring cell adenocarcinoma. The patient was treated with a chemotherapy regimen and palliative radiotherapy which led to improved performance status.

**Clinical discussion:**

Colon and rectum have different patterns of metastasis due to different vasculature and anatomy. Although metastatic bone lesions can be seen in both of them, talus bone involvement has been rarely reported. Non-specific skeletal-related events should be taken into consideration as they can be primary symptoms of bone metastasis or even the first symptoms that lead to CRC diagnosis.

**Conclusion:**

This case highlights a rare solitary metastatic pathway of CRC and underscores the importance of a comprehensive diagnostic work-up in patients with treatment-refractory bone disorders.

## Highlights

• This case reports a rare instance of solitary bone metastasis from (CRC) to the talus bone.
• Consider metastatic bone disease in patients presenting with non-specific orthopedic symptoms.
• Detailed imaging studies played a crucial role in identifying the bone metastasis.
• Highlights the importance of early diagnostic work-up in CRC patients with refractory bone conditions.

## 1 Introduction

CRC is one of the major global health concerns, the third leading causes of cancer-related mortality [1]. Metastasis remains a critical issue in treating of CRC despite significant advances in screening and therapeutic approaches [2]. Metastatic dissemination commonly involves the liver and lungs, but bone metastases occur in less than 7 % of cases [3]. Most CRC-related bone metastases affect the axial skeleton due to hematogenous dissemination through Batson's venous plexus [4]. Although bone metastases are less common, they often indicate a poor prognosis and add complexity to the treatment landscape [5]. Solitary bone metastases in CRC are rare, with fewer than 1 % of CRC patients presenting with such an occurrence [6]. Ankle metastases often present with non-specific symptoms such as pain, swelling, and decreased mobility, which can be mistaken for more common orthopedic conditions [7]. In this study we investigated the diagnostic challenges, therapeutic routes, clinicopathological features of solitary ankle metastases from CRC. The report adheres to the SCARE 2025 guidelines for case reports [8].

## 2 Case presentation

A 46-year-old male patient presented to our emergency center with the chief complaint of constipation for the past 6 days. The patient reported past medical history of right foot fracture sustained one year ago. On examination, his vital signs were within normal limits. Upon abdominal examination, distension and localized tenderness in the epigastric region and left lower quadrant were noted, with no rebound tenderness or guarding. The patient had a history of a right talus fracture sustained during exercise one year ago. Despite initial treatment with splinting, followed by cast immobilization and platelet-rich plasma (PRP) injections, the fracture did not heal, leading to further complications. At previous referral to another orthopedic clinic, the patient was managed as a candidate for avascular necrosis of the right talus. Laboratory investigation showed a hemoglobin level of 13.4 g/dL, a white blood cell counts of 10,000/μL, ESR of 10 mm/h, and an elevated C-reactive protein (CRP) level of 14 mg/L. Liver function tests, renal function tests, and electrolyte levels were within normal limits.

The abdominopelvic and thoracic CT scan showed a focal mass at the rectosigmoid junction, findings that are highly suggestive of a primary colonic neoplasm that indicate colon obstruction. Importantly, at this preliminary stage of evaluation, there were no overt imaging features such as abnormal nodal enlargement or secondary lesions in the liver, lungs, and other thoracoabdominal structures that would suggest metastatic dissemination. Based on the patient's clinical presentation and imaging findings, surgical intervention was indicated for a diagnosis of colonic obstruction.

Intraoperative findings have shown 10 cm obstructive rectosimoid junction mass without any seeding, ascites, liver, and other abdominal metastasis. The mass was excised, and a left hemicolectomy with T2 lymph node dissection and end colostomy (Hartmann's procedure) was performed.

Pathological examination revealed poorly differentiated signet ring cell adenocarcinoma, classified as Grade 3. Greatest dimension of tumor was 8 cm, with lymphovascular invasion (N2) and extension to the pericolorectal fat (T3). Immunohistochemistry (IHC) demonstrated positivity for cytokeratin 20 (CK20) and cytokeratin 7 (CDX2), and negative for cytokeratin 7 (CK7), synaptophysin, and chromogranin. The Ki-67 proliferation index was up to 70 %, reflecting a high mitotic rate.

**Fig. 1:**
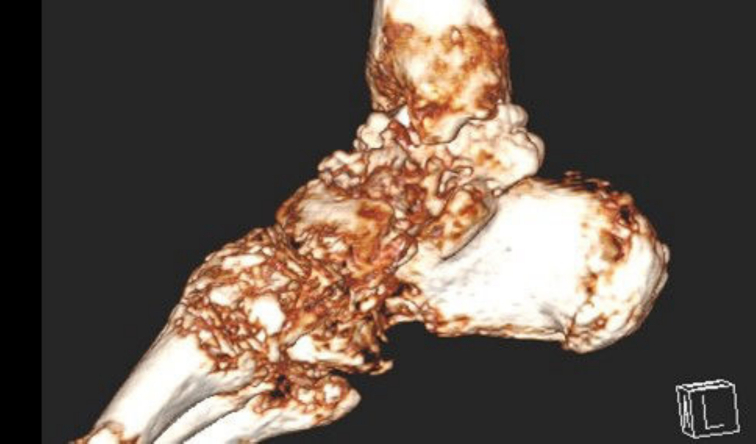
3D reconstruction CT scan of the ankle showing a lesion located in the right talus bone.

**Fig. 2:**
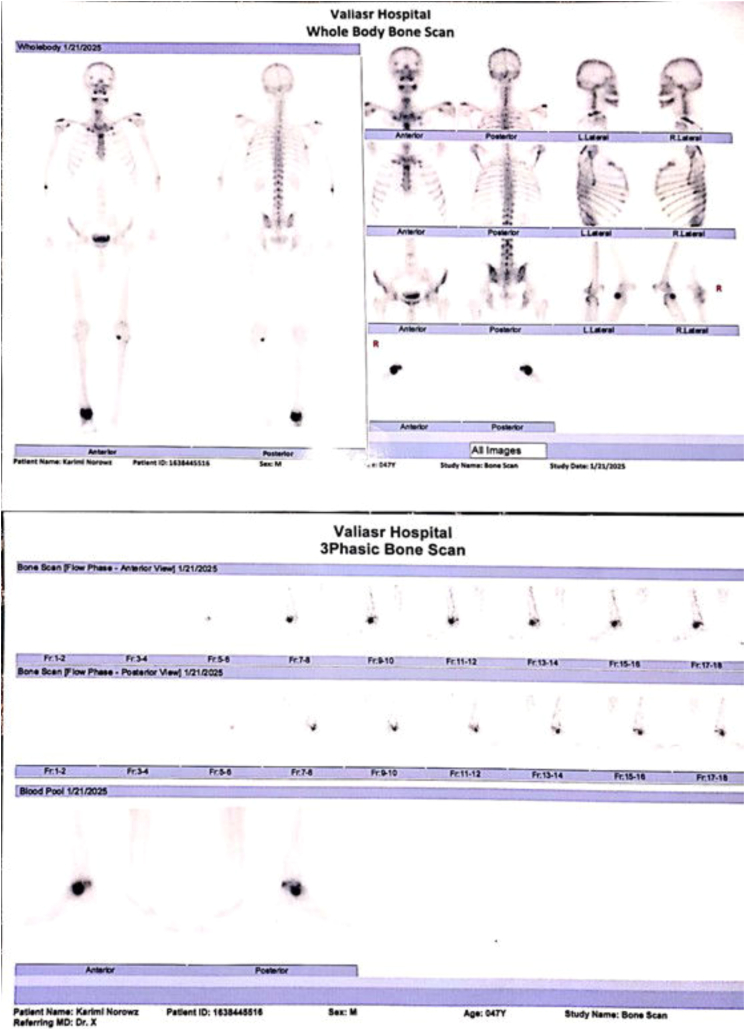
Triple-phase whole-body bone scan showing isolated, abnormal radiotracer uptake in the right ankle region. The flow and blood pool phases demonstrate hyperemia, while the delayed phase confirms a solitary metastatic lesion in the talus.

**Fig. 3:**
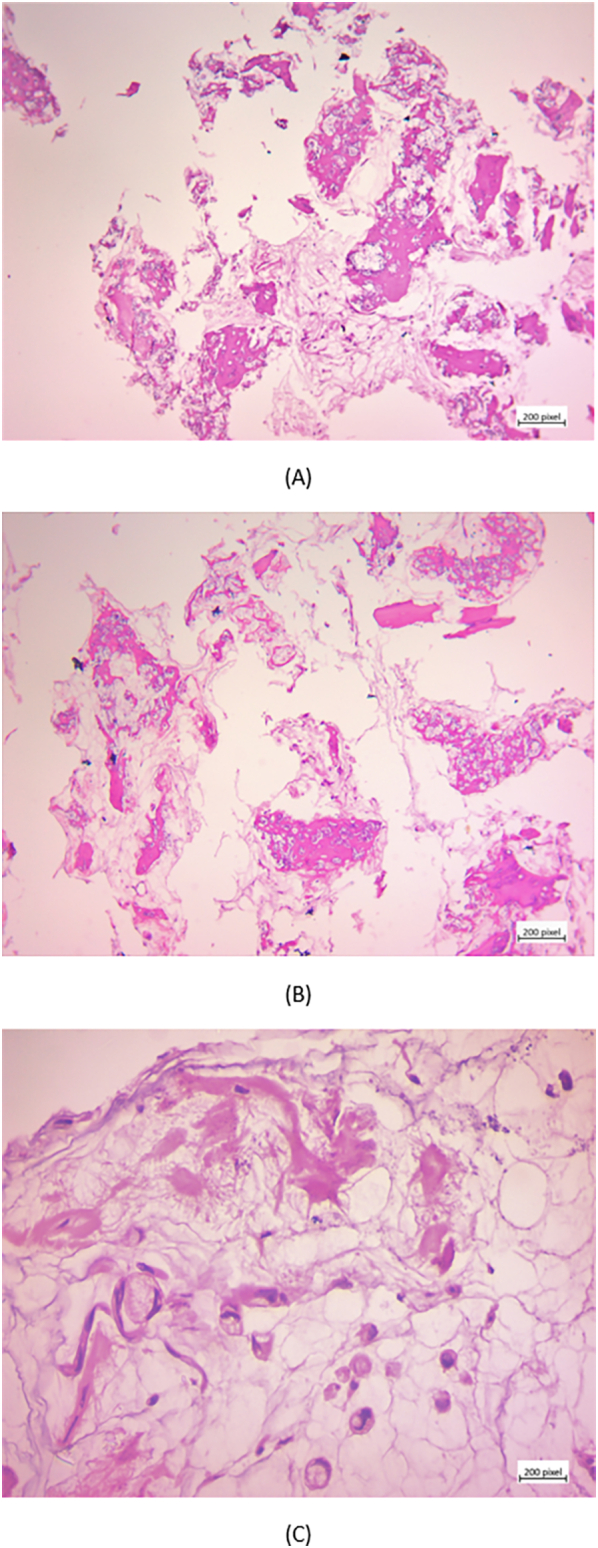
spongy bone filled with mucinous material and with floating new bone formations (×100, H&E) (A), extensive new bone formations and occasional signet-ring tumor cells (×100, H&E) (B), mucin-producing tumor cells floating in mucus-filled marrow spaces (×400, H&E) (C).

The patient was admitted to our orthopedic service for further investigation. The orthopedic service ordered a three-dimensional (3D) CT scan of the ankle and a whole-body bone scan for further evaluation. CT scan revealed a lesion located in the right talus bone (Fig. 1A), and the 3D Reconstruction images demonstrated irregular margins with evidence of cortical disruption, suggesting an aggressive, infiltrative process (Fig. 1B). Triple-phase whole-body bone scan (Fig. 2) illustrated hyper flow and increased blood pool throughout the right ankle region during the first and second phases. In the delayed (third) phase of the study, whole-body scanning revealed abnormal increased radiotracer uptake at the right ankle. Given these findings, image-guided biopsy of the talus lesion was undertaken to secure a definitive diagnosis. Histopathological examination of the talus lesion confirmed a mucin-producing tumor with signet ring cells, which had infiltrated the bone marrow, consistent with metastasis from the previously identified poorly differentiated colon adenocarcinoma (Fig. 3).

Following multidisciplinary consultation, chemotherapy regimen (5-fluorouracil + oxaliplatin) and palliative radiotherapy of right talus was initiated to the patient. After three cycles of chemotherapy and radiotherapy, the patient's performance status improved over time, and he reported enhanced ability to perform daily activities with minimal discomfort (Table 1).

**Table 1:** CRC with historical, clinical, and treatment variables.

First author (year)	Sex/Age	CRC history	Trauma history	Metastasis location	First symptom	CRC location	How long bone symptoms	Type of tumor	Bone radio therapy	Bone chemotherapy
Shamohammadi (2025)	M/46	0	1	Right talus	Ankle pain	Rectosigmoid junction	18 months	poor	1	1
Bakula (2023)^a^	F/78	0	0	Tibia	Ankle swelling	Rectum	17 days	moderate	0	1
Chalkidou (2009)^b^	M/77	1	0	Right tibia	Ankle pain	Rectum	1 year	NA	1	1
Ellington (2009)^c^	M/84	1	0	Right tibia and third metatarsal	Ankle swelling and pain	Colon	several months	NA	NA	NA
F. Kose (2008)^d^	F/42	1	0	Distal end of tibia	Ankle pain	Right colon	NA	NA	1	1
T.S.F.Tadross (2000)^e^	F/74	1	0	Distal left tibia	Ankle swelling and pain	Rectum	3 months	NA	1	0

Abbreviations: CRC: Colorectal Cancer/NA: Not Available/M: Male/F: Female.

a Bakula B, Karačić A, Stanić G, Romić I, Bakula M, Bogut A: Colorectal adenocarcinoma presenting with a pathological fracture due to a solitary bone metastasis to the tibia: a case report and literature review. *Gastroenterology Review/Przegląd Gastroenterologiczny* 2023, 18(1):115–122.

b Chalkidou AS, Boutis AL, Padelis P: Management of a Solitary Bone Metastasis to the Tibia from Colorectal Cancer. *Case Reports in Gastroenterology* 2009, 3(3):354–359.

c Ellington JK, Kneisl JS: Acrometastasis to the Foot:Three Case Reports With Primary Colon Cancer. *Foot & Ankle Specialist* 2009, 2(3):140–145.

d Kose F, Sakalli H, Sezer A, Mertsoylu H, Pourbagher A, Reyhan M, Ozyilkan O: Colon Adenocarcinoma and Solitary Tibia Metastasis: Rare Entity. *Journal of Gastrointestinal Cancer* 2008, 39(1):146–148.

e Tadross TSF, Checketts RG: Retrograde Intramedullary Nail for Metastatic Lesion of the Lower Tibia. *Foot & Ankle International* 2000, 21(8):683–685.

## 3 Discussion

The present case highlights the uncommon talus bone metastasis from primary CRC origin. Approximately 20 % of patients with CRC present with distant metastasis at the time of diagnosis [9,10]. Liver, lung, distant lymph nodes, and peritoneum are common sites of metastasis in CRC respectively [11]. However, bone metastasis is relatively uncommon and is associated with poor prognosis [9,12]. Spine is the most frequent site of bone metastasis in CRC, followed by pelvic bone and long bones [13]. The case presented in this study had metastatic lesion in talus bone and another case presented by Ellington JK et al. [14] had tibia and third bone involvement which are very rare sites of bone metastasis in CRC.

Rectal cancer exhibits a distinct metastatic pattern compared to colon cancer, largely driven by its unique anatomical and vascular features. Unlike colon cancer, where metastatic spread predominantly follows the portal circulation to the liver, rectal tumors can bypass hepatic filtration and disseminate directly to systemic sites [9,15]. The case reviewed in this study and three other cases presented by Bakula B et al. [6], Chalkidou AS et al. [16], and Tadross TSF et al. [17] had primary tumor in rectum/rectosigmoid junction and none of them had any other sites of metastases.

The diagnostic challenge posed by these atypical metastatic patterns is considerable. Patients may initially present with symptoms that mimic non-cancerous conditions. Common signs of bone metastasis are bone pain, pathologic fracture, spinal cord compression, and hypercalcemia [18]. In some cases, skeletal symptoms were the primary symptoms that led to CRC diagnosis. Bakula B et al. [6] presented a case with pretibial swelling followed by pathologic fracture. CRC was diagnosed after biopsy was taken from the bone lesion, in the absence of any significant bowel dysfunctions. Furthermore, ankle pain was precipitating other symptoms in the case reviewed in this study. Modalities such as CT scan, magnetic resonance imaging (MRI), and whole-body bone scan are critical for thorough staging and guiding subsequent management decisions. Furthermore, treatment strategies must be individualized. Generally, radiotherapy and bisphosphonates are commonly used as palliative therapy for bone pain [19,20].

## 4 Conclusion

Our case demonstrates the rare and atypical metastatic dissemination in CRC. The solitary ankle involvement, in the absence of other metastatic sites, raises suspicion of bone metastasis, especially in patients with treatment-refractory bone disorder. Local radiotherapy combined with chemotherapy in skeletal metastasis required for improve patient's quality of life, increase survival, and relieve pain.

## Abbreviations

CRC: Colorectal Cancer
CT: Computed Tomography
ESR: Erythrocyte Sedimentation Rate
CRP: C-Reactive Protein
PRP: Platelet-Rich Plasma
IHC: Immunohistochemistry
CK20: Cytokeratin 20
CK7: Cytokeratin 7
CDX2: Caudal Type Homeobox 2
PET-CT: Positron Emission Tomography-Computed Tomography
MRI: Magnetic Resonance Imaging

## Declaration of competing interest

The authors declared no conflicts of interest.
